# Regulation of p14^ARF ^expression by miR-24: a potential mechanism compromising the p53 response during retinoblastoma development

**DOI:** 10.1186/1471-2407-12-69

**Published:** 2012-02-15

**Authors:** Kwong-Him To, Sanja Pajovic, Brenda L Gallie, Brigitte L Thériault

**Affiliations:** 1 Campbell Family Institute for Cancer Research, Ontario Cancer Institute, University Health Network, Toronto, ON, Canada; 2 Toronto Western Hospital Research Institute, Ontario Cancer Institute, University Health Network, Toronto, ON, Canada; 3 Department of Molecular Genetics, University of Toronto, Toronto, ON, Canada; 4 Departments of Medical Biophysics and Ophthalmology, University of Toronto, Toronto, ON, Canada; 5 Department of Ophthalmology and Visual Science, Hospital for Sick Children, Toronto, ON, Canada; 6 Ontario Cancer Institute, University Health Network, 610 University Avenue, Toronto, ON M5G 2M9, Canada

## Abstract

**Background:**

Most human cancers show inactivation of both pRB- and p53-pathways. While retinoblastomas are initiated by loss of the *RB1 *tumor suppressor gene, *TP53 *mutations have not been found. High expression of the p53-antagonist MDM2 in human retinoblastomas may compromise p53 tumor surveillance so that *TP53 *mutations are not selected for in retinoblastoma tumorigenesis. We previously showed that p14^ARF ^protein, which activates p53 by inhibiting MDM2, is low in retinoblastomas despite high mRNA expression.

**Methods:**

In human fetal retinas, adult retinas, and retinoblastoma cells, we determined endogenous *p14^ARF ^*mRNA, ARF protein, and miR-24 expression, while integrity of p53 signalling in WERI-Rb1 cells was tested using an adenovirus vector expressing p14^ARF^. To study p14^ARF ^biogenesis, retinoblastoma cells were treated with the proteasome inhibitor, MG132, and siRNA against miR-24.

**Results:**

In human retinoblastoma cell lines, *p14^ARF ^*mRNA was disproportionally high relative to the level of p14^ARF ^protein expression, suggesting a perturbation of p14^ARF ^regulation. When p14^ARF ^was over-expressed by an adenovirus vector, expression of p53 and downstream targets increased and cell growth was inhibited indicating an intact p14^ARF^-p53 axis. To investigate the discrepancy between *p14^ARF ^*mRNA and protein in retinoblastoma, we examined p14^ARF ^biogenesis. The proteasome inhibitor, MG132, did not cause p14^ARF ^accumulation, although p14^ARF ^normally is degraded by proteasomes. miR-24, a microRNA that represses p14^ARF ^expression, is expressed in retinoblastoma cell lines and correlates with lower protein expression when compared to other cell lines with high *p14^ARF ^*mRNA. Transient over-expression of siRNA against miR-24 led to elevated p14^ARF ^protein in retinoblastoma cells.

**Conclusions:**

In retinoblastoma cells where high levels of *p14^ARF ^*mRNA are not accompanied by high p14^ARF ^protein, we found a correlation between miR-24 expression and low p14^ARF ^protein. p14^ARF ^protein levels were restored without change in mRNA abundance upon miR-24 inhibition suggesting that miR-24 could functionally repress expression, effectively blocking p53 tumor surveillance. During retinal tumorigenesis, miR-24 may intrinsically compromise the p53 response to *RB1 *loss.

## Background

Retinoblastoma is initiated by inactivation of both *RB1 *alleles encoding the protein, pRB that controls cell proliferation, differentiation and genomic stability. Persons heterozygous for a germline *RB1 *mutation are predisposed to cancer of the developing retina with a rate and penetrance unmatched in any other tissue. The unique sensitivity of developing retina to *RB1 *loss may be explained by an inability to trigger protective tumor suppressor pathways readily activated in other tissues.

As a safeguard against loss of pRB, cells generally respond by activating the potent tumor suppressor gene *TP53 *that induces apoptosis and cell cycle arrest [1]. p53 is tightly regulated by MDM2 and MDM4 through proteolysis and suppression of transactivating activity, respectively [2]. Crosstalk between the pRB and p53 pathways is mediated by p14^ARF ^(referred to as ARF hereafter). Loss of pRB specifically activates E2F1 to transcriptionally activate *ARF *through a novel response element that has activity in various tumor cells with defective pRB, but not in normally growing cells [3]. Once activated, ARF inhibits MDM2, leading to p53 stabilization and transcription of p53 target genes [4-6].

Concurrent inactivation of the pRB and p53 pathways is a hallmark of cancer. However in retinoblastoma, *TP53 *mutation has not been reported, and alternative modes of p53 inactivation implicating *MDM2 *and *MDM4 *have been suggested but remain to be confirmed, since the expression of *MDM2 *in retinoblastomas is similar to retina [7-9] and *MDM4 *is situated in a region of frequent genomic gain (chromosome 1q32) that also contains the oncogene *KIF14 *[10]. In addition, it has been suggested that retinoblastoma arises from a retinal cell that naturally expresses high level MDM2, thereby bypassing the requirement for p53 genetic inactivation [8].

We previously observed low ARF protein in retinoblastoma cell lines despite robust mRNA expression, suggesting protein instability or translational attenuation of ARF [7]. We now demonstrate repression of *ARF *expression by miR-24 in retinoblastoma that may effectively block activation of p53 tumor surveillance in response to *RB1 *loss.

## Methods

### Tissue culture

Cell lines were grown in a humidified 37°C incubator with 5% CO_2 _in their respective media with 100 U/ml penicillin and 0.1 mg/ml streptomycin (Wisent Bioproducts). Retinoblastoma cell lines with characterized *RB1 *mutations ([11,12] and manuscript in preparation) were grown in Iscove's MDM (Invitrogen) with 15% FetalClone III (HyClone), 10 mg/L insulin (Sigma-Aldrich) and 0.0004% (v/v) β-mercaptoethanol. HEK-293, HEK-293T (HEK-T), SKOV3 and HeLa were grown in DMEM-H16 with 10% FBS (Wisent Bioproducts). SaOS-2 were grown in DMEM-H21 with 10% FBS. OVCAR-3 was grown in RPMI-1640 with 20% FBS and 10 mg/L insulin. To study proteasome-mediated protein degradation, cells were incubated in 50 μM MG132 (Sigma-Aldrich) for 10 hours.

### RT-PCR

Total RNA was extracted using TRIzol (Invitrogen) according to manufacturer's instructions. RNA concentration and quality were determined using a Nanodrop-1000 spectrophotometer (Thermo Scientific). For cDNA synthesis, 1 μg of total RNA was reverse transcribed using random primers (Invitrogen) and Superscript II Reverse Transcriptase (Invitrogen). For gene expression analysis, PCR was performed using a RoboCycler Gradient 96 thermal cycler (Stratagene). PCR began with 2 minutes at 94°C, followed by 35 cycles of amplification, ending with 10 minutes at 72°C, in reaction mixture including 200 μM dNTPs, 2.5 mM MgCl_2_, 0.5 μM each of forward and reverse primers, 0.5 U Hot start DNA polymerase (Fermentas), 1× PCR buffer (Novagen) and 1 μl of cDNA product with a final volume of 25 μl.

### Quantitative real-time PCR

RNA extraction and cDNA synthesis were performed as described above. Real-time PCR was performed in a 7900HT Fast Real-Time PCR system using Universal PCR Master Mix in 384-well plates (Applied Biosystems). Taqman gene expression assays (Applied Biosystems) were employed to measure mRNA expression of *ARF *(Hs99999189_m1) and *GAPDH *(Hs99999905_m1) in triplicate. Mean relative gene expression and standard deviation were determined using the ΔΔCt method built-in to the SDS 2.2 software (Applied Biosystems). *GAPDH *was the endogenous control gene; fetal retina was the calibrator sample. Total RNA from fetal retina (pooled 16.5 and 17 weeks) was a kind gift from Dr. Rod Bremner, Toronto Western Research Institute, Toronto, ON. Adult retina (HR 50 and 108) was obtained from the enucleated eyes of cornea donors provided by the Eye Bank of Canada, with the University Health Network Research Ethics Board (REB) approval for anonymous use of discarded specimens.

### Western blot

Cell lines were dissociated mechanically in lysis buffer (phosphate buffered saline, 1% Nonidet P40, 5% sodium deoxycholate, 0.1% SDS, 1.0 μg/ml leupeptin, 0.1 mM PMSF, 1.0 μg/ml aproptinin and 100 μM sodium orthovanadate), lysed by three freeze-thaw cycles, incubated on ice for 30 minutes, and centrifuged to remove cellular debris. Protein lysates from human fetal (pooled 16.5 and 17 weeks) retinas were kindly provided by Dr. Rod Bremner. Adult retinas were provided by the Eye Bank of Canada and total protein lysates isolated as above. Protein concentration was determined using BioRad Protein Assay (BioRad). For SDS-PAGE, 50 μg of protein lysate was resolved in 4-20% Tris-Glycine gradient gels (Lonza) and transferred onto PVDF (BioRad) membranes. Membrane was blocked in 5% blotto (BioRad) in TBS overnight at 4°C and subsequently probed with primary antibody in TBS with 1% BSA and 0.05% Tween-20 at room temperature for one hour, followed by three washes in TBS with 0.1% BSA and 0.05% Tween-20. Primary antibodies and the respective dilutions used are as follows: ARF (1:500, 4 C6/4 clone, Cell Signaling Technologies, #2407 [13]), p21 (1:200, BD-Pharmingen, 556430), p53 (1:200, Santa Cruz, sc6243-G), BAX (1:200, Santa Cruz, sc7480), MDM2 (1:200, Calbiochem, OP115) and β-tubulin (1:1000, Sigma-Aldrich, T0198). Blots were incubated with primary antibodies, then secondary antibodies at specified dilutions in TBS with 1% BSA and 0.05% Tween-20 at room temperature for one hour, followed by three washes. Secondary antibodies were anti-rabbit-HRP (1:10000, Santa Cruz, sc2004), anti-goat-HRP (1:10000, Santa Cruz, sc2020) and anti-mouse-AP (1:10000, Santa Cruz, sc2008). Proteins were detected with HyGLO Chemilluminescence Detection Reagent (Denville). Proteins probed with AP-conjugated secondary antibodies were detected using NBT/BCIP (Denville).

### Immunostaining

Cells grown on a coverslip were incubated in 4% paraformaldehyde for 15 minutes at room temperature, followed by three washes in PBS, then incubated in 5% Triton-X for 10 minutes at room temperature. Blocking was performed for 30 minutes at room temperature in TBS with 10% DAKO Protein Block (DAKO-Cytomation), 1% BSA and 0.05% Tween-20. Slides were incubated with ARF primary antibody (1:200, Abcam, ab3642) in TBS with 1% BSA, 0.05% Tween-20 and 10% Antibody Diluent (DAKO-Cytomation), followed by three washes in TBS with 0.1% BSA and 0.05% Tween-20, then incubated with goat biotinylated anti-rabbit IgG secondary antibodies (1:200, Vector Laboratories, BA-1000) at room temperature for 1 hour, followed by three washes. ARF was detected with Streptavidin-Alexa-594 (Molecular Probes); nuclei were visualized with DAPI. Slides were mounted with DAKO-Cytomation Fluorescent Mounting Medium.

### Adenoviral-mediated ARF expression

E1/E3 early regions-deleted adenovirus encoding *gfp *(adgfp) or human *ARF *(adARF) [14] controlled by CMV promoter were kind gifts from Dr. Erik Knudsen (Kimmel Cancer Center, PA, USA) and Dr. Ruth Gjerset (Sidney Kimmel Cancer Center, CA, USA), respectively. To purify adenovirus, infected HEK-293 cells were resuspended in PBS with 10% glycerol, and viral particles were released by three freeze-thaw cycles; cellular debris was then removed by centrifugation. Viral titer was determined using QuickTiter Adenovirus Titer Immunoassay Kit (Cell Biolabs); adenovirus was stored at -72°C. To assess the effect of *ARF *overexpression on gene expression and cell growth, 200,000 WERI-Rb1 cells were seeded in 6-well plates pretreated with poly-D-lysine (Sigma-Aldrich) and incubated overnight. For immunostaining, cells were seeded on poly-D-lysine treated coverslips placed within 6-well plates. After overnight incubation, cells were rinsed once with PBS and incubated in 500 μl of serum-free Iscove's media containing adgfp or adARF at 20 multiplicity of infection (MOI) for 1 hour in a 37°C incubator with 5% CO_2_. Complete growth media (2 ml) was added to each well and incubated for four days, after which cells were harvested for gene expression analysis. Cell viability was determined by trypan blue exclusion (Invitrogen). Means and standard deviations were based on three replicates per treatment. Statistical significance was determined using two-tailed t-test.

### miRNA cDNA synthesis and quantitative real-time PCR

Total RNA extraction was performed as described above. A commercially available miRNA cDNA synthesis kit (TaqMan^® ^MicroRNA Reverse Transcription Kit, Applied Biosystems) was used to reverse transcribe miR-24 with specific RT primers (TaqMan^® ^miRNA Assays, Applied Biosystems). A sequence-specific assay (TaqMan^® ^real-time PCR assays, Applied Biosystems) was used to detect mature miR-24 from cell extracts. RNU44, a small non-coding RNA with wide and constant tissue distribution (Applied Biosystems), was used as the endogenous control.

### Anti-miR-24 siRNA

Asychronous WERI-Rb1 cells were transiently transfected with 50 pM of anti-miR-24 siRNA (Ambion, ID: AM12902) or control siRNA (Ambion, ID: AM17010) using a WERI-Rb1 cell transfection reagent (Altogen Biosystems) in serum-free Iscove's MDM. Cells were harvested for total protein 72 hours post-transfection and assayed for ARF and β-tubulin by Western Blot.

### Protein quantification

Signal intensity measurements for ARF and β-tubulin within each sample were calculated via Photoshop CS3. For each protein sample, the measure of ARF protein expression (pixel area multiplied by average pixel intensity) was normalized to β-tubulin expression, and then expressed as relative fold expression in comparison to normalized fetal retina ARF expression [15].

## Results

### Low ARF protein to mRNA expression ratios in human retinoblastoma cell lines

**Figure 1:**
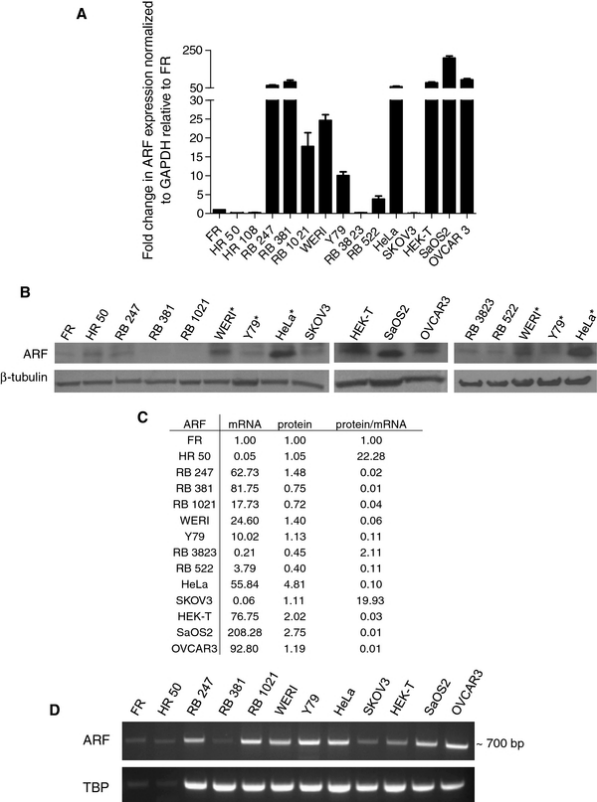
**Expression of *ARF *in human retinoblastoma cell lines**. (**A**) Comparison of *ARF *mRNA level between normal human fetal (FR) and adult retinas (HR 50 and 108), *RB1*^-/- ^retinoblastoma cell lines (RB 247, 381, 1021, WERI-Rb1 (WERI), Y79), *RB1*^+/+ ^cell lines (RB 3823, 522), and other cell lines (HeLa, SKOV3, HEK-T, SaOS2, OVCAR3) using quantitative real-time PCR. *GAPDH *was used as an endogenous control. (**B**) Western Blot analysis of ARF in fetal retina (FR), adult retina (HR 50), *RB1*^-/- ^retinoblastoma (RB247, 381, 1021, WERI-Rb1 (WERI), Y79), *RB1*^+/+ ^retinoblastoma (RB 3823, 522), and other (HeLa, HEK-293T, OVCAR-3 and SaOS-2) cell lines. *Same cell extracts were added to ensure consistent ARF protein expression/detection between immunoblots. (**C**) Ratio of relative ARF protein expression (normalized to β-tubulin, relative to FR) to relative *ARF *mRNA (normalized to GAPDH, relative to FR). (**D**) Confirmation of full length *ARF *mRNA expression in fetal retina (FR), human retina (HR 50), retinoblastoma cell lines and other cell lines using RT-PCR.

We have previously shown that ARF protein in primary retinoblastomas and cell lines is low compared to the level of mRNA [7], suggesting selective regulation of ARF expression during tumorigenesis. To elaborate on this finding, ARF expression was compared between normal human fetal (FR) and adult retinal (HR) tissues, retinoblastoma cell lines with characterized, non-functional *RB1 *[11,12] (*RB1*^-/-^), and cell lines known for high *ARF *mRNA expression (HeLa, HEK-T, SaOS-2 and OVCAR-3) or low *ARF *mRNA (SKOV3). Most *RB1*^-/- ^retinoblastomas (RB 247, 381, 1021, WERI-Rb1, Y79) expressed high levels of *ARF *mRNA, some (RB247, RB381) comparable to that of HeLa (Figure 1A). However, retinoblastomas with wild-type, intact *RB1 *(*RB1*^+/+^: RB 3823, RB 522) had very low levels of *ARF *mRNA. These *RB1^+/+ ^*retinoblastomas show no detectable mutations in the *RB1 *gene and express full-length pRB protein [16]. All retinoblastomas with the exception of WERI-Rb1 expressed low ARF protein relative to cell lines HeLa, HEK-T, SaOS2 and OVCAR3 (Figure 1B). The ratio of ARF protein to mRNA levels (normalized relative to FR) revealed that some *RB1*^-/- ^retinoblastoma cell lines (RB 247, RB 381, RB 1021, WERI-Rb1) demonstrate much lower ratios of protein:mRNA than both human fetal and adult retinas, *RB1*^+/+ ^cell lines (RB 3823 and 522), and cancer cell lines HeLa and SKOV3 (Figure 1C), indicating a defect in ARF protein translation. Since quantitative RT-PCR amplifies only 72 base pairs of the full length *ARF *mRNA, fragmented mRNA could be detected. RT-PCR spanning all three *ARF *exons confirmed the presence of intact, full length *ARF *mRNA (Figure 1D).

### Intact p53 signalling downstream of ARF

**Figure 2:**
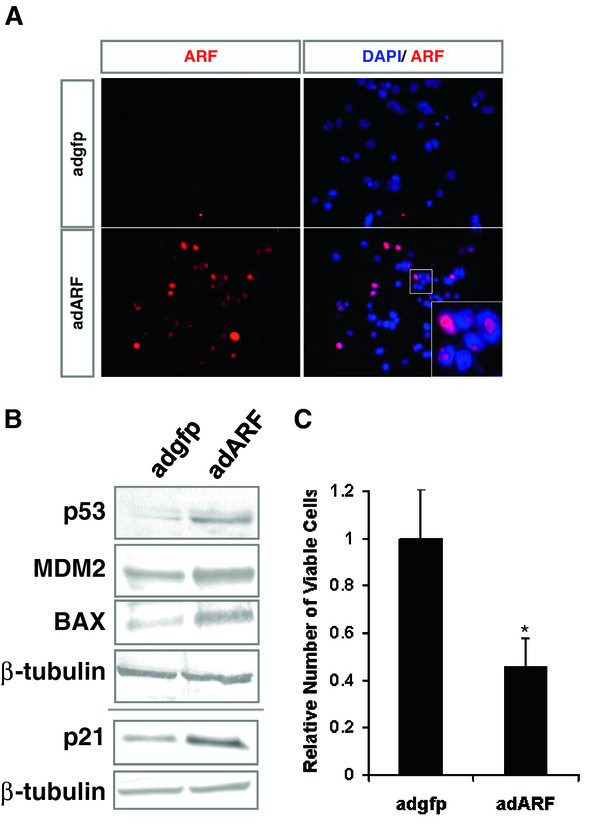
**Effect of exogenous ARF in retinoblastoma cells**. (**A**) Immunofluorescence (red) staining for ARF protein in the cell line WERI-Rb1 four days after infection with adenovirus encoding human ARF cDNA (adARF) or gfp (adgfp). Blue, DAPI stain. (**B**) Western Blot analyses of p53 and its transcriptional targets BAX, MDM2 and p21 in cells treated with adARF or adgfp four days after infection. (**C**) Relative number of viable cells after four days of treatment with adgfp or adARF. N = 3, t-test *P < 0.05.

To test if increased ARF can activate p53 and p53-transcriptional targets, adenovirus encoding human *ARF *cDNA (adARF) [14,17] was used to drive *ARF *expression in the retinoblastoma cell line WERI-Rb1, which expresses MDM2 and MDM4 [7,9]. After transduction, normal ARF nucleolar localization was detected as foci within the nucleus [18] (Figure 2A). AdARF-treated cells showed accumulation of p53 protein and p53-downstream targets p21, MDM2 and BAX (Figure 2B), and inhibition of cell growth (Figure 2C). Therefore, enforced expression of ARF activated p53 and p53-targets, and inhibited tumor cell growth, suggesting that optimal levels of ARF can activate p53 despite high MDM2 and MDM4 expression.

### Proteasomal degradation does not account for low ARF protein

**Figure 3:**
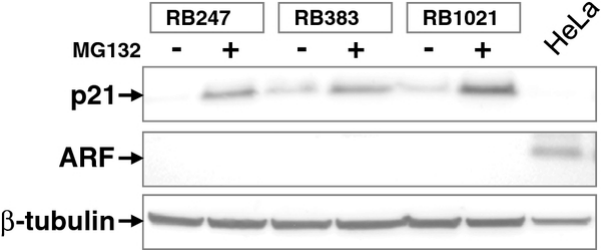
**Role of the proteasome in ARF regulation**. Western Blot analysis of ARF and p21 in retinoblastoma cell lines after MG132 treatment. Retinoblastoma cell lines were treated with the proteasome inhibitor MG132 for 10 hours. p21 was used as a positive control since its degradation is regulated by the ubiquitin-proteasome pathway.

ARF turnover is regulated by the ubiquitin-proteasome pathway through N-terminus ubiquitination [19]. To determine if ARF is aberrantly degraded by the proteasome, retinoblastoma cells were treated with the proteasome inhibitor, MG132. All treated cell lines responded with p21 accumulation, whose turnover is proteasome-regulated (Figure 3). Despite MG132 treatment, ARF remained undetectable in retinoblastoma cell lines compared to untreated HeLa cells. Therefore, aberrant proteasomal turnover does not account for low ARF protein in retinoblastoma, suggesting inhibition of ARF translation as a possible mechanism for low protein:mRNA ratios.

### High miR-24/ARF protein ratios in human retinoblastoma cell lines

**Figure 4:**
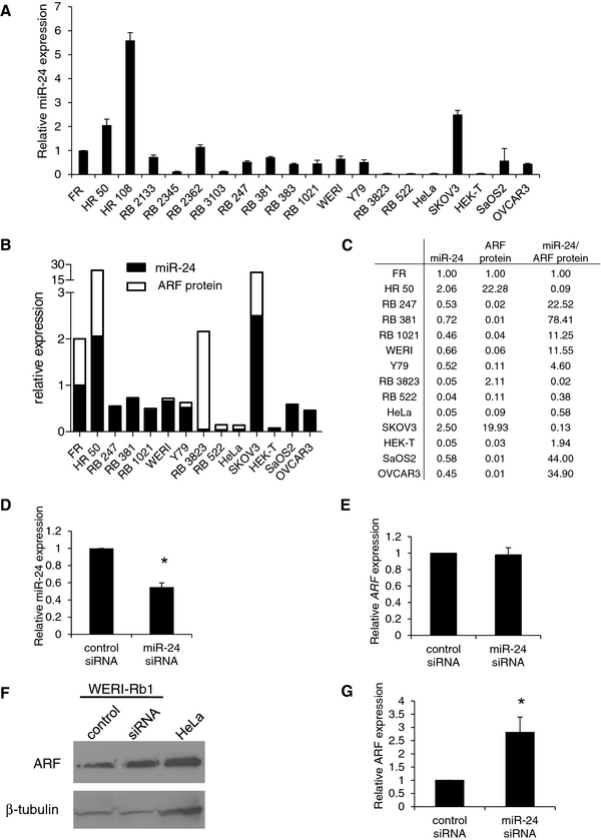
**Role of miR-24 in retinoblastoma ARF regulation**. (**A**) Real-time quantitative PCR analysis of miR-24 expression in human fetal retina (FR) and adult retina (HR 50 and 108), *RB1*^-/- ^retinoblastoma tumors (RB 2133, 2345, 2362, 3103), *RB1*^-/- ^retinoblastoma cell lines (RB 247, 381, 383, 1021, WERI-Rb1 (WERI), Y79), *RB1*^+/+ ^retinoblastoma cell lines (RB 3823, 522) and other cell lines (HeLa, SKOV3, HEK-T, SaOS2, OVCAR3). N = 3. (**B**) Comparison of normalized ARF protein expression (values taken from ARF protein/*ARF *mRNA expression, as depicted in Figure 1C) to miR-24 expression in human fetal retina (FR), human adult retina (HR 50), *RB1^-/- ^*retinoblastoma cell lines (RB 247, 381, 1021, WERI-Rb1 (WERI), Y79), *RB1*^+/+ ^retinoblastoma cell lines (RB 3823, 522) and other cell lines (HeLa, SKOV3, HEK-T, SaOS2, OVCAR3). (**C**) Ratio of miR-24 expression (normalized to RNU44), relative to normalized ARF protein expression (values taken from ARF protein/*ARF *mRNA expression, as depicted in Figure 1C). (**D**) Quantitative real-time PCR analysis of miR-24 after WERI-Rb1 cells were transiently transfected with anti-miR-24 siRNA or control siRNA for 72 hours, and presented as fold change normalized to RNU44 expression, relative to control siRNA; N = 3, t-test *P < 0.05. (**E**) Quantitative real-time PCR analysis of *ARF *mRNA after anti-miR-24 siRNA or control siRNA transfection, N = 3. (**F**) ARF expression measured by immunoblot in response to control siRNA and anti-miR-24 siRNA transient transfection of WERI-Rb1; ARF protein levels in HeLa cells are shown for positive ARF protein expression. (**G**) Graph represents fold change in ARF protein in WERI-Rb1 cells normalized to β-tubulin, relative to control siRNA. N = 3, t-test *P < 0.05.

The microRNA miR-24 [20], expressed in both normal retinas and retinoblastomas [17], regulates the 3' untranslated region of both *p16^INK4a ^*and *ARF *mRNA [20]. AdARF mRNA did not contain the two potential miR-24 target sites, which may explain the accumulation and activity of exogenously-expressed ARF (Figure 2). Analysis of miR-24 in human fetal and adult retinas, primary retinoblastoma tumors, retinoblastoma cell lines and other cell lines revealed the highest miR-24 expression in human adult retinas, consistent with reports of elevated miR-24 expression in terminally differentiated cells [21]. However, fetal retinas show in general higher levels of miR-24 compared to the average expression of primary retinoblastomas and retinoblastoma cell lines (Figure 4A). All *RB1^-/- ^*cell lines, in addition to two out of the 4 primary *RB1^-/- ^*retinoblastomas (RB 2133 and RB 2362) demonstrated higher miR-24 expression than the *RB1^+/+ ^*cell lines (Figure 4A).

To evaluate the potential role of miR-24 expression in retinoblastoma and other cell lines, miR-24 expression was compared to the level of mRNA-normalized ARF protein expression (taking into account the protein/mRNA ratios from Figure 1C). Comparisons of normalized miR-24 expression and ARF protein expression demonstrates that *RB1*^-/- ^cell lines have very high miR-24 relative to the amount of expressed protein, in comparison to *RB1*^+/+ ^cell lines with low ARF protein and miR-24 (RB 3823 and 522; Figure 4A,B and 4C). Other cell lines with abundant ARF protein (HeLa, HEK-T) but low miR-24 (Figure 4B and 4C), or high miR-24 and ARF protein (SKOV3), demonstrate lower miR-24:protein ratios, suggesting that high miR-24 levels could functionally regulate ARF protein expression in *RB1*^-/- ^cell lines.

### miR-24 suppresses ARF in retinoblastoma cell lines

To further assess the function of miR-24 in *RB1*^-/- ^retinoblastoma cell lines, we transiently transfected WERI-Rb1 cells with a small interfering RNA (siRNA) against miR-24. Knockdown of miR-24 (approximately 50%, Figure 4D) resulted in increased ARF protein (2.8-fold), but no change in *ARF *mRNA (Figure 4E, F &4G). Thus, low ARF protein in retinoblastomas despite robust mRNA expression, at least in part, may reflect the presence and activity of miR-24 to inhibit ARF expression.

## Discussion

We demonstrate that most human retinoblastomas in which *RB1 *is inactivated (*RB1*^-/-^) show low ARF protein despite high *ARF *mRNA. In contrast, *RB1*^+/+ ^retinoblastoma tumors show low *ARF *mRNA. This data confirms previous findings [3] and our results in the Rb knockout mouse (data not shown) that loss of pRB leads to an increase in *ARF *mRNA due to transcriptional activation by E2F1. High *ARF *mRNA but low ARF protein also suggests attenuation of synthesis or instability of ARF in most *RB1*^-/- ^retinoblastoma cell lines. For the cell line Y79, low ARF was at least in part due to low *ARF *mRNA. Although Y79 has an *RB1*^-/- ^genome, a truncated pRb protein is produced; this may affect its own transcription and interplay with E2F family members [22,23].

Through viral overexpression of ARF, we showed in WERI-Rb1 cells that: i) exogenous ARF protein expression induces the p53 response even when MDM2 and MDM4 are expressed; and ii) the ARF-induced p53 response results in a functional decrease in cell viability. These data demonstrate that the integrity of the p53 pathway is maintained in retinoblastoma cells, and that ARF protein expression may play an important role in controlling p53 responses. We have shown that proteasomal degradation does not account for low ARF protein in the WERI-Rb1 cell line, thus other mechanisms of ARF protein regulation may exist. Indeed, miR-24 has documented expression in normal retinas and retinoblastomas, and demonstrated translational repression of *p16^INK4a ^*mRNA, which shares 100% homology with the 3' untranslated region of *ARF *[20,24,25]. In our expression analyses, miR-24 is less abundant in human retinoblastomas than in normal fetal and adult retinas, with highest expression in adult retinas, consistent with reports of elevated miR-24 expression in terminally differentiated cells [21]. We showed that fetal retinas in general demonstrate higher miR-24 expression than retinoblastoma tumors. This data suggests regulation of miR-24 expression during retinal development, perhaps due to its role in regulation of p53 via ARF to suppress p53 hyperactivation and unwanted cell death. Indeed miR-24 was shown to be involved in developmental apoptosis in Xenopus retina [26]. Paradoxically, when pRB is inactivated during retinal tumorigenesis, ARF protein regulation mediated by high miR-24 intrinsic levels may impede the tumor suppressor functions of p53. Moreover, the pleiotropic miR-24, in addition to its regulation of ARF, has been shown to repress expression of MYC and E2F2 [21], which are important in driving proliferation and imperative to tumor growth. The reduced miR-24 level in retinoblastomas relative to normal retinas may be explained by the requirement of sustained expression of proliferation genes in tumor cells while maintaining sufficient amounts of miR-24 to repress the ARF tumor suppressor. Intriguingly, the low miR-24:protein ratios in *RB1*^+/+ ^retinoblastomas are consistent with the notion that, in the absence of selection pressure from *ARF *activation induced by *RB1 *loss, tumor cells may maximize proliferation through the derepression of MYC and E2F2 [21] without the compromise of tumor suppressor activation. Thus the observed miR-24 in retinoblastoma tumors might be at an optimal level that maximizes tumor cell growth and survival. Some *RB1^-/- ^*primary tumors showed low miR-24 expression, comparable to *RB1^+/+ ^*cell lines. These "low miR-24" *RB1^-/- ^*tumors raise the possibility that the level of miR-24 expression may be insufficient to compromise the p53 response in some retinoblastoma tumors. However other factors besides expression, such as components of the RISC complex, may influence the effect of miR-24 activity in retinoblastoma cells [27]. *ARF *could also be regulated by other microRNAs in retinoblastomas. For example, expression of miR-125b and miR-24 is similar in retinoblastomas [28], and miR-125b has been shown to regulate the 3' untranslated region of *p16^INK4a ^*and *ARF *mRNA [20]. To gain insight on the role of miR-24 on the ARF-p53 axis, a thorough examination of the biological and functional implications of miR-24 expression in human retinal and retinoblastoma tumor development is thus warranted.

Regulation of ARF protein expression by miR-24 in retinoblastoma cell lines points to a possible mechanism through which ARF protein is decreased in retinoblastoma cells. We uncovered an intact p53 response in WERI-Rb1 cells through overexpression of ARF protein; however, whether targeting miR-24 in retinoblastoma cells would increase ARF protein expression to optimal levels to elicit a p53 response remains to be investigated.

Our miR-24 and ARF protein expression data are in agreement with ARF protein regulation by miR-24 that is unique to *RB1*^-/- ^retinoblastomas. However, SaOS2 and OVCAR3 cell lines show similar miR-24:protein ratios as *RB1*^-/- ^retinoblastoma cell lines, suggesting that miR-24 regulation of ARF may apply to other cell types. Given that the other cell lines tested display different genetic or protein changes to those seen in retinoblastoma cell lines, such as wild-type *RB1 *(SKOV3 and OVCAR3), inactivated pRb (HeLa and HEK-T) or mutant *TP53 *(SKOV3, OVCAR3 and SaOS2) [29,30], further investigation into the functional mechanisms of ARF regulation via miR-24 are required. Differential miR-24:protein ratios between *RB1*^-/- ^retinoblastomas, fetal retina and adult retina, and *RB1*^+/+ ^retinoblastomas, combined with ARF-mediated apoptosis and miR-24-regulated ARF expression in WERI-Rb1 cells nonetheless demonstrate a unique mechanism in *RB1*^-/- ^retinoblastoma tumors through which proliferation could be maintained by miR-24 suppression of ARF.

## Conclusions

Our data indicates that p53-tumor surveillance in response to *RB1 *loss may be suboptimal in the developing retina, not only due to high levels of MDM2 and MDM4, but inadequate levels of ARF, possibly as a result of suppression by miR-24. We demonstrated that exogenous ARF expression activated p53 and its downstream targets in retinoblastoma cells, resulting in reduced proliferation. We suggest that low endogenous ARF protein may present an innate mechanism that could regulate p53 functions during retinoblastoma development.

We propose that the unique retinal sensitivity to tumorigenesis induced by *RB1 *loss is primarily due to a barrier to apoptosis inherent in developing retina, consistent with the demonstration in mice that the cell of retinoblastoma origin is intrinsically death resistant [31]. This mechanism may facilitate the control of developmental apoptosis to achieve precise numbers of various cell types in the retina, reflected by high MDM2 expression in the developing retina [8]. However, when retinal cells are transformed, high MDM2 expression is consistently expressed, but at variable levels [7]; thus other factors may affect p53 tumor surveillance. We suggest that miR-24-mediated repression of *ARF*, a crucial signal transducer that bridges loss of pRB to p53-tumor surveillance, may also compromise the proper response to *RB1 *inactivation, alleviating the requirement for genetic abrogation of the p53-pathway during retinoblastoma development. Our results point to the potential use of ARF supplementation in the development of therapeutic approaches targeting p53 activation in retinoblastoma.

## Competing interests

The authors declare that they have no competing interests.

## Authors' contributions

KHT, SP, BLG and BLT conceived and designed the study. BLT performed the miR-24 expression analysis and anti-miR-24 siRNA experiment, and KHT performed all other experiments. All authors contributed to the writing and editing of this paper. All authors read and approved the final manuscript.

## Pre-publication history

The pre-publication history for this paper can be accessed here:

http://www.biomedcentral.com/1471-2407/12/69/prepub
